# The value of local malaria strains for serological studies: local strains *versus* Palo Alto reference strain

**DOI:** 10.1186/s12936-015-0734-z

**Published:** 2015-05-31

**Authors:** Fode Diop, Gora Diop, Makhtar Niang, Babacar Diouf, Daouda Ndiaye, Vincent Richard, Aissatou Toure Balde

**Affiliations:** Immunology Unit, Institut Pasteur Dakar, Dakar, Senegal; Laboratory of Parasitology-Biology, Faculty of Sciences and Technology, Cheikh Anta DIOP University of Dakar, Dakar, Senegal; Immunogenetic Unit, Institut Pasteur Dakar, Dakar, Senegal; Laboratory of Parasitology-Mycology, Faculty of Medicine and Pharmacy, Cheikh Anta Diop University of Dakar, Dakar, Senegal; Epidemiology Unit, Institut Pasteur Dakar, Dakar, Senegal

## Abstract

**Background:**

The standardization of the type of crude *Plasmodium falciparum* extracts for assays to evaluate the overall anti-blood-stage immune response in humans may be beneficial to malaria pre-elimination programmes. However, there is no consensus on which strain is appropriate for routine analyses. This study aimed to compare the responses of malaria IgG antibodies in serum collections from Dielmo and Ndiop to crude extracts of merozoites and schizonts of local and reference strains of *P. falciparum*.

**Methods:**

Malaria antibodies were evaluated using serological tests for exposure to three local strains (0703, F15 and F16) and the *P. falciparum* reference Palo Alto strain (PA). A total of 218 sera collected in 2000 from inhabitants of the villages of Dielmo and Ndiop were used: 142 from Dielmo and 76 from Ndiop. The biological collection was used to evaluate by ELISA the prevalence of IgG antibodies against crude merozoite and schizont extracts. The genetics of the local and reference strains were compared.

**Results:**

There was genetic divergence between strains 0703, F15, F16 and PA. IgG responses against local and reference strains correlated well (0.6 to 0.8; *p* < 0.01). Ig G responses were highest to schizont and merozoite extracts from the field strain of *P. falciparum* 0703 adapted to *in vitro* culture. Extracts of *P. falciparum* strain 0703 isolated from a subject in Dielmo was the most widely recognized [91.3 % (199/218) and 81.2 % (177/218) for schizonts and merozoites, respectively], although the responses were high for merozoites from PA [85.3 % (186/218)] the reference strain, and the two strains isolated from subjects living in Dakar: F15 [90.4 % (197/218)] and F16 [72.5 % (158/218)].

**Conclusions:**

For serological studies, the local strain provided the most complete picture of exposure to transmission and malaria prevalence in the local context. However, for the standardization of this method by different laboratories, the reference strain appeared to perform sufficiently well to be used for the evaluation of malaria control programmes.

## Background

Programmes of pre-elimination of malaria have been underway in Senegal since 2010, and the burden of malaria has decreased substantially [1–3]. The changes in the epidemiology nevertheless need to be monitored with effective tools that allow changes in patterns of transmission to be documented. In the context of pre-elimination, serological markers are promising tools for detecting exposure to malaria and evaluating malaria control efforts. This is particularly true for areas where transmission has decreased to levels below the detection limit of microscopy and where assessing transmission intensity directly by determining the exposure to malaria-infected mosquitoes (entomological inoculation rate [EIR]) has become difficult [4, 5]. Serological markers have been suggested to be fully informative indicators of the dynamics of malaria transmission [6, 7]. Various methods based on characterized antigens have been used to measure specific humoral responses to malaria and to evaluate transmission intensity [6, 8]. One of the limitations of these methods is that the immune response to a given antigen may depend on genetics, and be affected by polymorphisms [9]. However, responses to parasite crudes extracts, which are mixtures of numerous different antigens, are less sensitive to these constraints [9, 10]. Crude merozoite and schizont lysates have been used to explore the response to a wide panel of parasite antigens and material derived from parasite lysates has been used to detect antibodies specific to *Plasmodium falciparum* blood stages [11, 12]. Antibody responses to crude extracts of schizonts of a local *P. falciparum* strain reflected the changes of malaria profiles due to various interventions [13]. However, the antigenic content of such extracts is poorly defined and may vary, depending upon the type of crude extract and the strain used [12]. These issues complicate the comparison of seroepidemiological analyses conducted in different laboratories and the interpretation of results. There is currently no consensus on the type of crude extract, or strain, or *P. falciparum* antigen to be used in standard assays for the evaluation of the overall anti-blood-stage immune response in humans.

This work aimed to compare merozoite and schizont extracts from local and reference strains to identify the most suitable extract for routine analysis of antibody responses to *P. falciparum* blood stages in malaria-exposed individuals.

## Methods

### Populations and serum collection

Since 1990, the Dielmo project was established between the Institut Pasteur of Dakar, the Institute for Research and Development, the Senegal Ministry of Health and the University of Dakar. This project was initially approved by the Ministry of Health of Senegal and the assembled village population. The project aim was to study the natural history of malaria and more specifically the determinants of protection and biological markers associated to protection/exposure through longitudinal follow up of the inhabitants of the villages of Dielmo and Ndiop [14]. For this purpose clinical, entomological and parasitical studies were performed and blood samples were collected for immunological studies and stored in a biobank.

Written informed consent from individuals enrolled in the project and parents or guardians of children who provided blood samples was obtained. The consent to participate to the project was regularly renewed. Approval was obtained from the National Ethics Committee for Health Research of Senegal and ad-hoc committees of the Ministry of Health, the Pasteur Institutes (Dakar and Paris), and Institute for Research and Development did audits regularly.

This study is based on the samples stored in the biobank and is in line with the objectives of the Dielmo project [14].

This comparative analysis study was conducted using sera collected in 2000 from inhabitants of the villages of Ndiop and Dielmo. A total of 220 sera from individuals less than 20 years old were used: 76 from Ndiop and 141 from Dielmo. In 2000, Ndiop and Dielmo had differences in malaria endemicity and contrasting contexts of transmission: in Ndiop, transmission was seasonal with an entomological inoculation rate of 80 infectious bites/person/year, whereas in Dielmo the transmission was continuous with 482 infectious bites/person/year [15]. Blood samples were collected before the end of the rainy season in 2000 when transmission was high in both Ndiop and Dielmo [16]. The sera were separated from blood cells and stored at −20 °C until use for immunological studies.

### Crude antigen extracts

Four parasite strains (0703, F15, F16 and PA) were used in this study and continuously cultured on O^+^ erythrocytes in RPMI containing 0.5 % Albumax using the candle jar method [17]. Three field strains of *P. falciparum* isolated from clinical or asymptomatic malaria-infected Senegalese patients and adapted for *in vitro* culture as described elsewhere [13, 18]. The 0703 strain was isolated from an inhabitant of Dielmo on November 1991 and successfully adapted to continuous *in vitro* culture thereafter [19]. F15 and F16 strains were isolated in Dakar on February 2010 and March 2010, respectively, from patients of the medical analysis laboratory and were also successfully adapted to continuous *in vitro* culture. The Palo Alto Uganda reference strain FCR3 (PA, Uganda) had been isolated from a Ugandan patient [20] and had been routinely cultured for years in the laboratory. Strains were cultured separately at different time periods to avoid cross contamination between strains. Extracts of schizonts of *P. falciparum* were prepared as previously described [12, 18]. Briefly, for schizont extracts, after centrifugation, one volume of the pellet was lysed in three volumes of sterile distilled water and vortexed for homogenization; the extracted schizonts were frozen without further centrifugation in liquid nitrogen in working aliquots. The merozoite extract was prepared from synchronous parasites as described by others [12, 21]. In brief, merozoites were collected by stepwise centrifugation of culture supernatants at 2,000 and 4,000 g*,* washed three times in sterile phosphate buffered saline (PBS), counted, and frozen in liquid nitrogen.

### DNA extraction and genotyping

Genomic DNA was extracted from wild field Senegalese isolates of *P. falciparum* adapted in continuous culture (0703, F15, F16) and from the PA reference strain using a QiAmp ® DNA Blood MiniKit (Qiagen Catalogue # 51106) according to the manufacturer’s recommendations. Nested PCR genotyping was performed for the variable block 3 region of *msp-2*, considered to be the most informative genetic marker for assessment of *P. falciparum* genetic diversity [22]. The initial PCR amplification with primers pairs flanking the *msp-2* (block 3) region was followed by individual nested PCR reactions using family-specific primers for *msp-2* (FC27 and 3D7/IC), using previously described standard protocols [23]. The PCR conditions and primers sequences were as described by Snounou *et al.* [24, 25]. The PCR-amplified *msp-2* products were separated by electrophoresis on 1.5 % agarose gel and the fragments were visualized under UV transillumination with ethidium-bromide. The sizes were estimated by comparison with a 100 bp (Hyperladder, Bioline®) molecular weight marker.

### ELISA assay

ELISA Maxisorp plates (Nunc, Roskilde, Denmark) and a dose effect assay using positive control sera were used to determine the optimal dilution of the extracts to use in the assay. The optimal dilution found for each extract was used for coating the plates. Plates were coated with 100 μl of water-soluble extract for all crude extracts. Non-infected red blood cell extract was prepared in the same conditions, incubated overnight at 4 °C and washed. Then, 200 μl of a blocking buffer (2 % BSA in PBS with 0.05 % Tween) was added to each well and the plates were incubated for 1 h at 37 °C. Plasma samples were diluted at 1/200 in a dilution buffer (1 % BSA in PBS with 0.05 % Tween) and 100 μl of diluted serum was distributed into each well. Negative and positive controls were included on each plate: positive controls were from Dielmo/Ndiop hyperimmune individuals and negative controls were from European individuals. Polyclonal goat anti-human IgG conjugated to peroxidase at a dilution of 1/6,000 in the dilution buffer (1 % BSA in PBS-Tween 0.05 %) was then added. Bound peroxidase was detected with ortho-toluidine /H_2_O_2_ (100 μl) and the reaction was stopped by addition of 4 N H_2_SO_4_ (50 μl/well). Between each incubation phase, the ELISA plates were washed extensively with PBS-0.05 % Tween. For each assay, plasma samples were tested in duplicates on the same ELISA plate. The optical density (OD) at 450 nm was read in a BIO-RAD Microplate Reader (iMark) [12]. The final OD value of each serum tested was obtained by calculating the mean OD of the duplicates. Sera from negative controls were used to define the cutting-off value for positivity based on an optical density of 2 fold the mean optical density (OD) of these negative controls. Results was expressed as OD ratio calculated as mean OD test/mean OD neg. and a ratio OD test/mean OD neg >2 was defined as cutting-off value for positive response. Inter-assay variations for positive controls did not exceed 20 %. The reproducibility of this technique has been found satisfactory with coefficients of variation of 4 % by using two positive controls (two different pools of hyperimmune serums), 10 negative controls and the same crude extract preparation on each plate [12, 26].

### Statistical analysis

*R* software was used for statistical analysis. Non parametric tests (Kruskal Wallis, Wilcoxon) and the Spearman test were used to study non-parametric quantitative variables. Fisher’s exact test was used for qualitative variables. Differences were considered statistically significant for *p* < 0.05 [27].

## Results

### Genotyping analysis

**Fig. 1:**
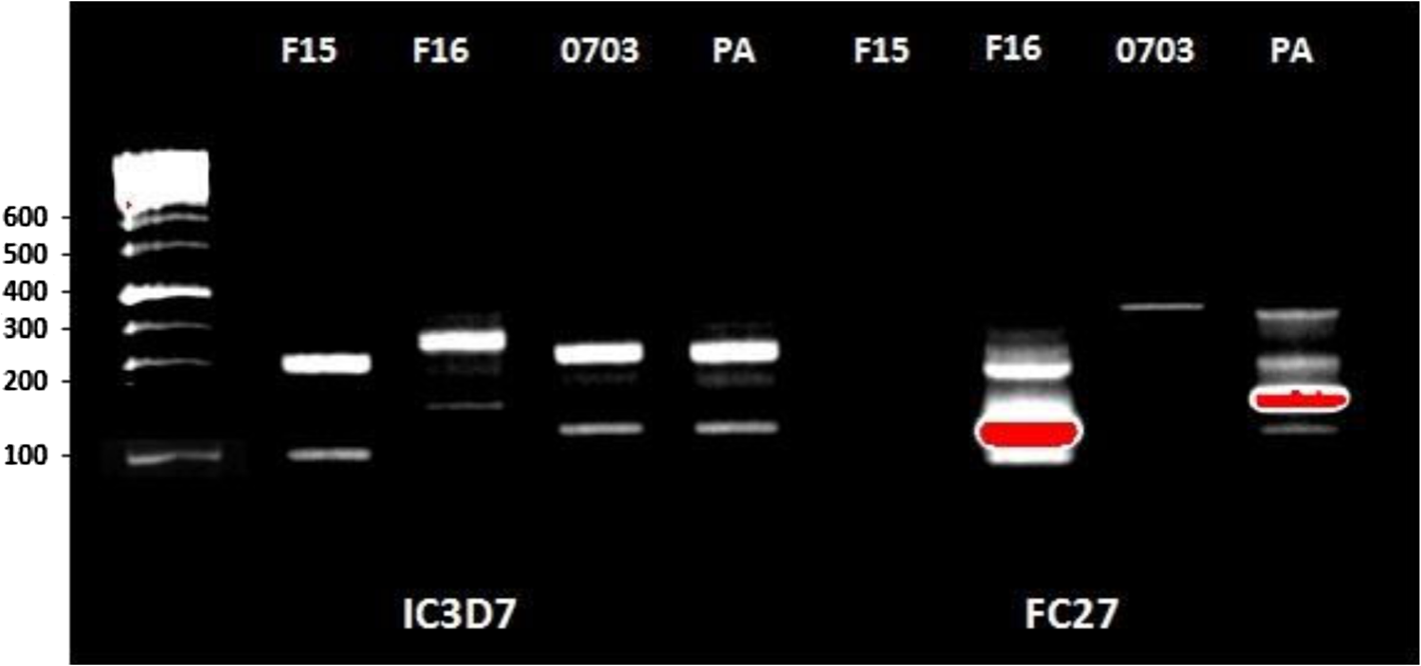
PCR analysis of the polymorphic region of *msp2* (block 3) of different *P.falciparum* isolates from Dielmo (0703), Dakar (F15 and F16) and Uganda (PA). The DNA size marker is a 100 bp ladder

Allelic diversity between the four parasite strains (F15, F16, 0703 and PA) was determined by assessing length polymorphism within the *msp2* locus (Fig. 1). The two allelic types of *msp2* were unequally distributed within the different parasite strains tested. Although two predominant IC3D7 allelic types were detected in all parasites strains, the number of FC27 allelic types differed between the different strains. The two most frequent IC3D7 alleles harboured by strain F16 were unique and different from those detected in the other parasite strains. For the FC27 allele family, no amplification product was detected for strain F16 whereas one, two and four bands were detected for 0703, F16 and PA, respectively. The single FC27 allele detected in strain 0703 was absent from all other parasite strains. The genotypes identified in the F16 strain were different from those of PA. These findings clearly demonstrate that the different parasite strains used in this study were genetically divergent.

### Correlation between antibody levels to crudes extracts

**Fig. 2:**
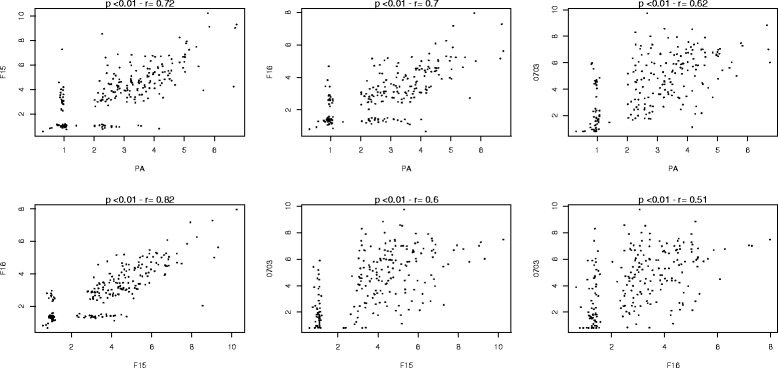
Correlation between Ab responses to schizont crude extracts

**Fig. 3:**
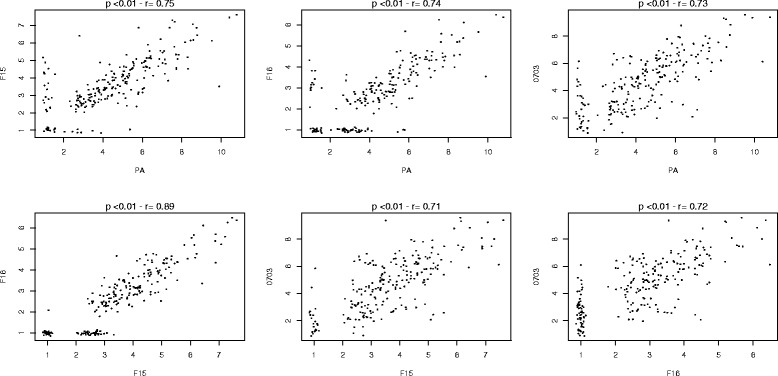
Correlation between Ab responses to merozoite crude extracts

The immune responses to the various strains were analysed by Spearman correlation. Individual antibody responses to crude PA, F15 and F16 schizont extracts were positively and significantly correlated with the antibody levels to the 0703 schizont extract (*rho* values of 0.62, 0.6, and 0.56 respectively for PA, F15 and F16; *p* < 0.01) (Fig. 2). There was a strong and positive correlation between antibody levels against crude F15, F16 and PA schizont extracts (*rho* values ranged from 0.7 to 0.8; *p* < 0.01). The OD ratios obtained with IgG against PA, F15 and F16 merozoites was also significantly correlated with OD ratios obtained for 0703 merozoites (*rho* values of 0.73, 0.71, and 0.72 respectively for PA, F15 and F16; *p* < 0.01) (Fig. 3).

### Comparison of antibody levels against crude schizont extracts

**Fig. 4:**
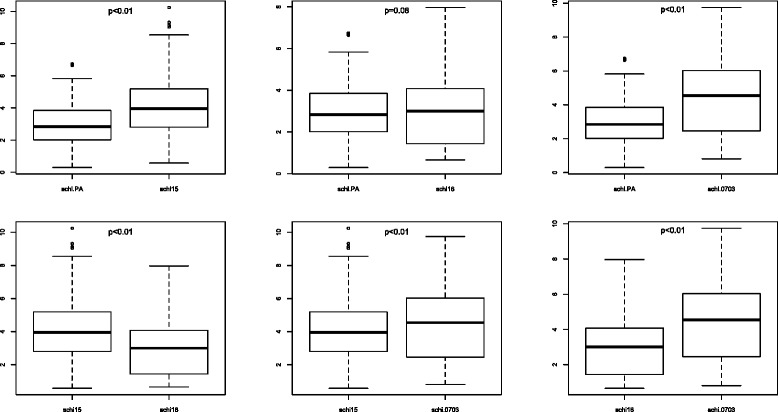
Comparison of antibody responses against schizont crude extracts (PA [Palo Alto], F15 [Foundation Pasteur *P. falciparum* 15], F16 [Foundation Pasteur *P. falciparum* 16], 0703 [*P. falciparum* 0703]. Measurements were performed with sera collected in 2000 from inhabitants of the villages of Dielmo and Ndiop

The mean antibody responses measured as OD ratios were 2.86 for schiPA, 3.89 for schiF15, 3.01 for schiF16 and 4.34 for schi0703 (Fig. 4). The Kruskal Wallis test indicated that there was significantly less total IgG against schiPA than against 0703 and F15, but that it was not significantly different to that against F16. The distribution of anti *P. falciparum* antibodies indicated that significantly higher IgG absorbance values were obtained using crude schi0703 extract than other extracts, with an overall *p* < 0.01.

### Comparison of antibody levels against crude merozoite extracts

**Fig. 5:**
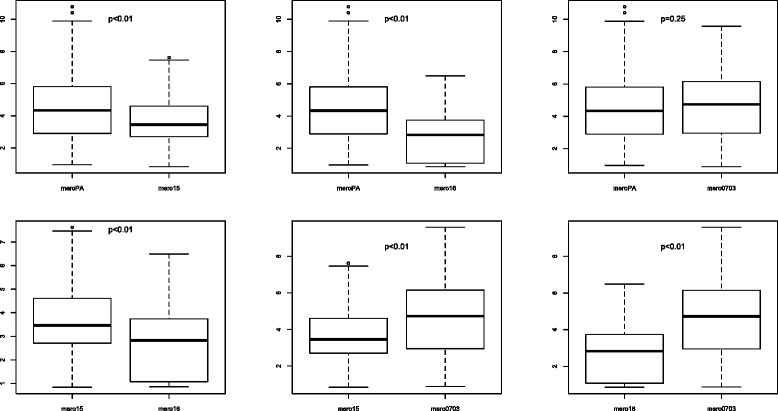
Comparison of antibody responses against merozoite crude extracts. (PA [Palo Alto], F15 [Foundation Pasteur *P. falciparum* 15], F16 [Foundation Pasteur *P. falciparum* 16], 0703 [*P. falciparum* 0703]. Measurements were performed with sera collected in 2000 from inhabitants of the villages of Dielmo and Ndiop

Mean antibody responses, expressed as OD ratios were 4.44 for meroPA, 3.63 for meroF15, 2.79 for meroF16 and 4.64 for mero0703 (Fig. 5). The value for IgG against mero0703 was significantly higher than other values except for meroPA for which the difference was not significant. The antibody response against meroF16 was significantly lower than those against other strains (*p* < 0.01). The response against meroPA was significantly higher than those against meroF15 and meroF16 (*p* < 0.01).

### Cross comparison of antibody levels against crude schizont and merozoite extracts

**Fig. 6:**
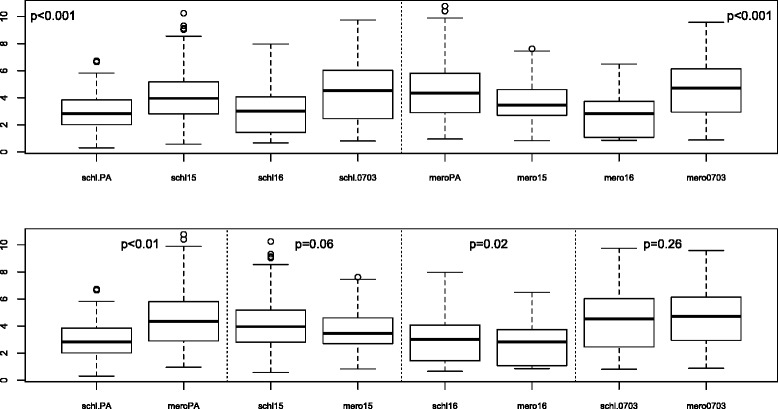
Cross comparison of antibody responses between *Plasmodium falciparum* schizonts and merozoites*.* (PA [Palo Alto], F15 [Foundation Pasteur *P. falciparum* 15], F16 [Foundation Pasteur *P. falciparum* 16], 0703 [*P. falciparum* 0703]. Measurements were performed with sera collected in 2000 from inhabitants of the villages of Dielmo and Ndiop

Antibody responses against schizont extracts and merozoite extracts were determined and compared (Fig. 6). The mean levels of antibody responses against merozoites were significantly higher than those against schizonts for the strains PA and F16 (*p* < 0.01), but there was no difference for strains F15 (*p* = 0.06) and 0703 (*p* = 0.26). The mean level of the antibody response against 0703 was higher than those of the other strains although the difference with meroPA was not significant.

### Percentages of positive responders against crude merozoite and schizont extracts

**Fig. 7:**
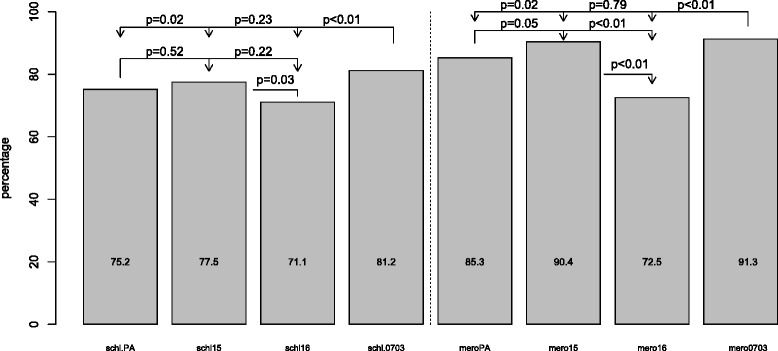
Positive responders to crude extracts of four *Plasmodium falciparum* strains (PA [Palo Alto], F15 [Foundation Pasteur *P. falciparum* 15], F16 [Foundation Pasteur *P. falciparum* 16], 0703 [*P. falciparum* 0703]. Measurements were performed with sera collected in 2000 from inhabitants of the villages of Dielmo and Ndiop

The percentages of positive responders (OD ratio > 2) to merozoite and schizont crude extracts were compared (Fig. 7 and Table 1). There was significant differences for reference strain PA merozoite extract (85.3 %) *vs.*75.2 % for schizont extract but not for strains F16 (72.5 % *vs.* 71.1 %), F15 (90.4 % *vs* 77.5 %) and 0703 (91.3 % *vs.* 81.2 %) (Table 1). The percentage of positive responders to mero16 (72.5 %) was significantly lower than to meroPA (85.3 %), mero15 (90.4 %) and mero0703 (90.4 %). However, there was no difference in the frequency of responders to F15 and 0703 (Fig. 7). The percentage of positive responders against schi0703 (81.2 %) was significantly higher than those to PA (75.2 %) and F16 (71.1 %) strains but not different to the value for schiF15 (77.5 %).

**Table 1:** Percentage of positive responders to crude extracts of four *Plasmodium falciparum* strains

Strains	Antigens	
	Schizonts (%)	Merozoites (%)
PA	75.2 (164/218)	85.3 (186/218)
F15	77.5 (169/218)	90.4 (197/218)
F16	71.1 (155/218)	72.2 (158/218)
0703	81.2 (177/218)	91.3 (199/218)

Measurements were performed with sera collected in 2000 from inhabitants of the villages of Dielmo and Ndiop

*PA* Palo Alto, *F15* Foundation Pasteur *P. falciparum* 15, *F16* Foundation Pasteur *P. falciparum* 16, *0703 P. falciparum* 0703

## Discussion

The study reported here involved an analysis, by an Enzyme Linked ImmunoSorbent Assay approach, of IgG responses to extracts of merozoites and schizonts from local and reference strains; the aim was to identify the most suitable extract for routine analysis of the levels of antibodies to *P. falciparum* blood stages in individuals exposed to malaria.

Samples from immune individuals from Dielmo and Ndiop were tested for their reactions to crude extract of four *P. falciparum* strains. The *mps2 g*enotype was used here to compare the parasite strains included in the study. Various genotypes were identified, based on PCR fragment size, revealing genetic diversity of the parasite strains included [25].

The IgG responses obtained with the four strains were strongly and positively correlated. The strong and positive correlation between strains PA, F15, F16 and 0703 for IgG antibody levels observed indicates that any of these crude extracts could be used to determine total anti-*P. falciparum* antibody responses in malaria endemic areas. The correlation between local and the reference strain means that laboratories could use reference strains for routine analysis; this is an advantage because adapting and maintaining wild parasite strains in continuous *in vitro* culture is complicated, and often unsuccessful [28].

Immunoassays using crude extracts from local strains as antigens are useful for evaluating immunity but, often, require the adaptation of *P. falciparum* isolates to continuous *in vitro* cultivation [13, 19]. To date, no clear consensus exists on which strain or what type of *P. falciparum* extract (merozoites or schizonts) to use for serological studies. There is a need for a standard assay, in particular for inter-laboratory comparisons of immune responses against strains from different origins.

The high IgG values observed in response to schizonts and merozoites of strain 0703, the local strain isolated in Dielmo and Ndiop, are consistent with the study population’s contact with this local strain, and the higher prevalence of positive responders against merozoites and schizonts confirmed the good recognition for this strain. These observations are in agreement with previous studies [13]. A high level of antibody recognition of strains PA, F15, and F16 was also obtained in samples from both Dielmo and Ndiop villages. This finding is consistent with studies conducted in Mali, Gambia, Cambodia and Peru [29, 30].

Although there was a significantly higher signal detected for IgG responses to 0703 than to other strains, notably F15, the frequencies of responders were less different: 81.2 % (177/218) vs 77.5 % (169/218) and 91.3 % (199/218) vs 90.4 % (197/218) for schizont and merozoite extracts, respectively. Therefore, it is not clear whether the high signal reflects a better IgG reactivity to a geographically-matched isolate or an immune response strongly elicited by more frequent inoculation. But whatever the origin of this higher signal the fact that frequencies of responders are less different between strains leads to the conclusion that for standardization using the parameter of frequency of responder as an indicator of malaria dynamics could be a better choice. This point raises the important issue of the cutting-off value to adopt for defining positivity. In this study the cutting-off chosen was values of OD ratio (mean sera OD/mean controls OD) > 2. This is an arbitrary value but, in the authors’ opinion, this value is high enough to exclude very low titers which could be difficult to interpret. Another method of defining positivity could be to adopt for positivity values > mean OD of controls + three standard deviations. This leads to higher number of positive responders as in these assays the standard deviations of controls are very low.

In high to moderate transmission areas as those studied here very low cut-off values would not be suitable for following malaria transmission dynamics as it could be not enough sensitive. But in areas where malaria transmission is very low and where elimination is envisaged, lower cut-off than ratio > 2 could probably be though.

The greater recognition of strain 0703 suggests that the use of a local strain may allow optimal determination of total anti-falciparum Ab responses in an area. These observations are also relevant to issues about the malaria indicators used in serological studies and some of the incoherencies related to the crude extract at different scales in dings for the level and prevalence of antibodies revealed no difference between merozoites and schizonts of the local strain [30]. Therefore, schizont extracts may be more appropriate for laboratories as preparation of crude extracts of antigens is more straightforward from schizonts than from merozoites.

The findings reported herein indicate that, for the analysis of serological data, distinctions should be made between local and reference strains. The use of a reference strain should help with obtaining homogeneous and comparable data from different laboratories in different study areas and be useful for monitoring. However, local strains may be valuable for detecting low level infections in local context, although further work is needed to confirm this possibility.
